# Soft drink consumption and liver fibrosis in type 2 diabetes

**DOI:** 10.3389/fnut.2025.1726040

**Published:** 2025-12-18

**Authors:** Arianna Ferro, Martina Bollati, Gian Paolo Caviglia, Angelo Armandi, Stefania Bellini, Selene Limoncelli, Giulio Mengozzi, Federica Barutta, Fabio Broglio, Guglielmo Beccuti, Elisabetta Bugianesi, Marilena Durazzo, Gabriella Gruden

**Affiliations:** 1 Department of Medical Sciences, University of Turin, Turin, Italy; 2 Division of Endocrinology, Diabetes and Metabolism, Azienda Ospedaliera Universitaria (AOU) Città della Salute e della Scienza di Torino, Turin, Italy; 3 Division of Gastroenterology and Hepatology, Azienda Ospedaliera Universitaria (AOU) Città della Salute e della Scienza di Torino, Turin, Italy; 4 Clinical Biochemistry Laboratory “Baldi e Riberi”, Azienda Ospedaliera Universitaria (AOU) Città della Salute e della Scienza di Torino, Turin, Italy; 5 Division of Internal Medicine 3, Azienda Ospedaliera Universitaria (AOU) Città della Salute e della Scienza di Torino, Turin, Italy

**Keywords:** lifestyle, liver fibrosis, liver stiffness, MASLD, soft drink, sugar-sweetened beverage, type 2 diabetes

## Abstract

**Introduction:**

Metabolic dysfunction–associated steatotic liver disease (MASLD) is highly prevalent among individuals with type 2 diabetes mellitus (T2DM), and liver fibrosis represents its strongest predictor of adverse outcomes. Soft drinks (SDs), a major source of added sugars and fructose, have been linked to metabolic disorders, but evidence on their relationship with liver fibrosis in patients with T2DM is limited. This study investigated the association between SDs consumption and liver fibrosis in adults with both T2DM and liver steatosis.

**Methods:**

We analyzed 273 participants from the TESEO-DM cohort with imaging-documented hepatic steatosis (Controlled Attenuation Parameter, CAP ≥248 dB/m). SDs intake was assessed using the validated EPIC food frequency questionnaire and categorized as rarely/never, 1–4 servings per month, or >1 servings per week. Liver stiffness measurement (LSM) was assessed using vibration-controlled transient elastography and LSM >7 used as cut-off to define significant liver fibrosis.

**Results:**

In age- and sex-adjusted linear regression, SDs intake was directly associated with LSM (β = 0.181, 95% CI: 0.062–0.299, *p* = 0.003). The association remained significant after adjustment for diabetes duration, total caloric intake, high-density lipoprotein cholesterol, and either body mass index (β = 0.153, 95% CI: 0.032–0.274, *p* = 0.014) or CAP (β = 0.150; 95% CI: 0.028–0.274; *p* = 0.017). In logistic regression, participants consuming >1 SDs per week had increased odds of significant liver fibrosis (OR: 3.77, 95% CI: 1.33–10.66) compared with those rarely or never consuming SDs independent of age, sex, diabetes duration, and obesity. Inclusion into the model of tertiles of CAP in place of obesity did not modify the results (OR: 3.11 95% CI: 1.09–8.86).

**Conclusions:**

These findings suggest that even modest soft drink consumption is independently associated with higher liver stiffness in individuals with T2DM and liver steatosis, supporting recommendations to limit added sugar intake for liver health.

## 1 Introduction

Metabolic dysfunction–associated steatotic liver disease (MASLD), formerly referred to as nonalcoholic fatty liver disease (NAFLD), is the most common chronic liver disorder worldwide and a leading cause of mortality (1–4). It encompasses a spectrum of hepatic injury ranging from simple steatosis to steatohepatitis (MASH—metabolic dysfunction-associated steatohepatitis) with increasing degree of fibrosis (3, 5). Among these, liver fibrosis is the strongest predictor of clinical outcomes, being closely associated with advanced liver disease, hepatocellular carcinoma, and both liver-related and cardiovascular mortality (2, 6). Therefore, the identification of modifiable risk factors for fibrosis represents a major priority for prevention and early intervention (3, 5).

MASLD is tightly linked to metabolic dysfunction. Obesity, metabolic syndrome, and type 2 diabetes mellitus (T2DM) are among its most important risk factors (1, 5). In fact, MASLD prevalence has been reported to range between 65.3 and 73.6% among individuals with T2DM (7, 8). Moreover, both the presence and duration of T2DM are major determinants of fibrosis progression in MASLD (9).

Soft drinks (SDs) are a prominent component of modern dietary patterns and represent a major source of added sugars. They are also rich in fructose, which promotes hepatic gluconeogenesis and *de novo* lipogenesis, thereby favoring hepatic fat accumulation (10). SDs consumption has been associated with a wide range of adverse health outcomes (11–13), including NAFLD (14). A prospective cohort study demonstrated a clear dose–response relationship, with consumption of four or more servings per week associated with a 45% increased risk of incident NAFLD (15). Also, a case-control study found an association between SDs consumption and liver steatosis independently of metabolic syndrome diagnosis (16). Furthermore, a short-term clinical trial among adolescent boys aged 11–16 years showed that reducing free sugar and fructose intake significantly decreased liver fat compared with a usual diet (17).

While these findings support an indirect role of SDs in fibrosis development—primarily through obesity and steatosis—evidence for a direct relationship between SDs and liver fibrosis in MASLD remains limited (18). Importantly, data in high-risk populations, such as individuals with T2DM, are still lacking.

In this study, we aimed to investigate the association between SDs consumption and liver fibrosis in a cohort of patients with T2DM and MASLD.

## 2 Materials and methods

### 2.1 Study design and population

The study included individuals with T2DM who were consecutively and prospectively enrolled between July 2019 and February 2025 as part of the ongoing TESEO-DM (Traguardi di Eccellenza nelle Scienze mediche Esplorando le Omiche - Diabete Mellito) cohort, which investigates chronic complications of T2DM. Eligible participants were adults (≥18 years) referred for the first time to the Unified Diabetes Center at San Giovanni Antica Sede Hospital (Turin). Upon enrollment, detailed demographic and clinical data were collected, including age, sex, physical activity, alcohol and tobacco use, cardiovascular risk factors, chronic diabetes complications, and current pharmacologic treatments. The European Prospective Investigation into Cancer and Nutrition (EPIC) and the Mediterranean Diet Score (MDS) questionnaire was administered to explore nutritional habits by a single qualified dietitian (19, 20). All participants underwent a complete physical examination with evaluation of anthropometric parameters, fasting blood sampling for biochemical analyses, and vibration controlled transient elastography (VCTE) with assessment of controlled attenuation parameter (CAP; FibroScan^®^530, Echosens, Paris, France). Additional assessments included fundus oculi examination, 12-lead electrocardiogram, and transthoracic echocardiography.

**Figure 1:**
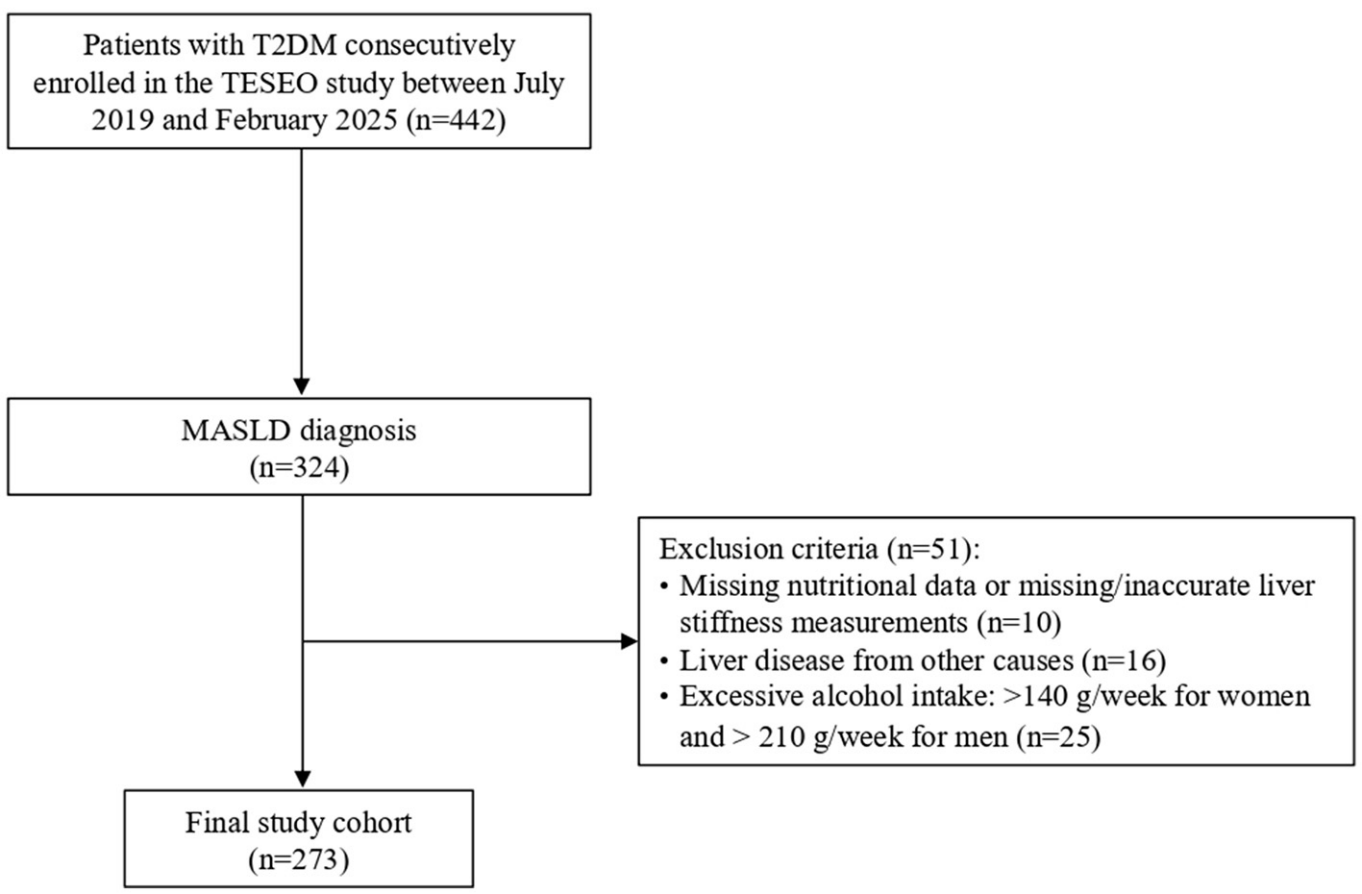
Participant selection flow diagram. MASLD, metabolic dysfunction-associated steatotic liver disease; T2DM, type 2 diabetes mellitus.

The initial TESEO-DM cohort comprised 442 adult patients with T2D of whom 324 had a diagnosis of MASLD (21) and were therefore selected for further analysis. A total of 273 participants were ultimately included in the analysis (Figure 1) after applying the following exclusion criteria: missing data on soft drink consumption, missing or inaccurate liver stiffness measurement, presence of other known causes of liver disease (e.g., viral hepatitis, autoimmune hepatitis, hemochromatosis, Wilson's disease, alpha-1 antitrypsin deficiency, or drug-induced injury). Alcohol abuse was defined as a self-reported ethanol intake >140 g per week for women and 210 g per week for men. The study protocol was approved by the local Ethics Committee, and all participants provided written informed consent prior to enrollment.

### 2.2 Biochemistry

Glycated hemoglobin (HbA1c) was measured using an immunoenzymatic assay and standardized to the Diabetes Control and Complications Trial (DCCT) reference system. Total cholesterol, triglycerides, high-density lipoprotein (HDL) cholesterol, aspartate aminotransferase (AST), alanine aminotransferase (ALT), and serum creatinine were determined using automated enzymatic assays on a Cobas-Bio analyzer.

### 2.3 Definitions and calculations

Physical activity was assessed using the short version of the International Physical Activity Questionnaire (IPAQ-short) (22), with total weekly energy expenditure calculated as metabolic equivalent tasks (METs) × minutes of activity (MET-min/week) and classified as low (< 700), intermediate (700–2,519), or high (>2,519). Participants were categorized into three groups based on smoking status: current smokers, former smokers, and never smokers. Body mass index (BMI) was calculated as weight in kilograms divided by height in meters squared (kg/m^2^). General obesity was defined as a BMI ≥30 kg/m^2^. Blood pressure (BP) was measured using a standard manual sphygmomanometer (Hawksley, Lancing, UK). Hypertension was defined as systolic BP ≥130 mmHg and/or diastolic BP ≥80 mmHg, confirmed on at least two separate visits, or current use of antihypertensive medications (23). Low-density lipoprotein cholesterol (LDL-C) was estimated using the Friedewald formula. Estimated glomerular filtration rate (eGFR) was calculated from serum creatinine using the CKD-EPI formula. Chronic kidney disease (CKD) was defined as eGFR ≤ 60 ml/min/1.73 m^2^ (24). Diabetic retinopathy was evaluated by a specialist ophthalmologist using retinal images acquired with the Optomed Aurora device (Midimedical). Retinopathy was classified as absent or present and the more severely affected eye was used for classification. Coronary heart disease (CHD) was defined as a documented history of myocardial infarction, angina pectoris, percutaneous coronary intervention (PCI), or coronary artery bypass grafting (CABG). Participants showing clinical, electrocardiographic, or echocardiographic signs suggestive of CHD were classified as CHD patients if the diagnosis was confirmed by further evaluation.

### 2.4 Frequency of soft drinks intake and Mediterranean diet

Dietary habits were assessed using the validated food frequency questionnaire developed within the EPIC study (19). The questionnaire assessed SDs consumption during the year preceding the interview, with frequencies ranging from “never or < 1 serving per month” to “≥6 servings per day.” For analytical purposes, participants were categorized into four groups: never/rarely, 1–4 servings/month, >1 serving/week, >1 serving/day. Average daily caloric intake was estimated using the FETA EPIC software (25).

Adherence to the Mediterranean diet was evaluated using the 14-item Mediterranean Diet Score (MDS) questionnaire (20), a self-administered tool with binary (yes/no) responses across key dietary components. One point was assigned for each affirmative response, with higher total scores indicating greater adherence to the Mediterranean dietary pattern.

### 2.5 Assessment of liver status

Vibration-controlled transient elastography (VCTE) with controlled attenuation parameter (CAP) was performed after an overnight fast to assess liver stiffness and hepatic steatosis. Examinations were conducted with participants in the supine position, the right arm extended above the head, and the probe (M or XL, according to body habitus) placed in the right intercostal spaces. For each subject, at least 10 valid measurements were obtained, and the median value was recorded. Only technically reliable results, defined as an interquartile range (IQR)/median < 30%, were included in the analysis (26). Liver stiffness was expressed in kilopascals (kPa) and CAP in decibels per meter (dB/m). Significant liver fibrosis was defined as LSM >7.0 kPa, while hepatic steatosis was defined as CAP ≥248 dB/m (5).

### 2.6 Statistical analysis

Continuous variables are presented as mean ± standard deviation (SD) when normally distributed, and as median with interquartile range (IQR) when non-normally distributed. Categorical variables are reported as absolute numbers and percentages. Normality was assessed using the Shapiro–Wilk and Kolmogorov–Smirnov tests. Between-group comparisons of continuous variables were performed using two-tailed Student's *t*-tests for independent samples or ANOVA, as appropriate. Non-normally distributed variables were log-transformed prior to analysis. Categorical variables were compared using the Chi-square test or Fisher's exact test, as appropriate.

Univariate and multivariate linear regression analyses were performed to evaluate the association between liver stiffness measurement (LSM, dependent variable) and increasing categories of SDs intake (independent variable), adjusting for relevant covariates: age, sex, diabetes duration, total caloric intake, HDL, and either BMI or CAP tertiles. Due to collinearity between BMI and CAP, separate models were fitted for each variable.

Logistic regression models were used to assess the association between SDs intake categories and the odds of clinically significant liver fibrosis, defined using two clinically relevant cut-offs (>7 kPa and ≥8 kPa). Multivariable models were adjusted for age, sex, diabetes duration, obesity, and categories of SDs intake.

All statistical tests were two-sided, and a *p*-value < 0.05 was considered statistically significant. Analyses were conducted using SPSS Statistics, version 29.0.2.0 (IBM Corp., Armonk, NY, USA).

## 3 Results

### 3.1 Study population

Table 1 summarizes the demographic, anthropometric, and clinical characteristics of the 273 participants included in the study. The mean age was 61.04 ± 8.1 years, with a slight greater prevalence of women. Average adherence to the Mediterranean diet was relatively high. However, only 9.2% reported high levels of physical activity, and 18.7% were current smokers. Obesity was present in 70% of participants, while nearly all had arterial hypertension (93.4%). The cohort was characterized by a relatively short duration of diabetes and generally good glycemic control. The prevalence of diabetes-related complications was 10.3% for CKD, 6.6% for diabetic retinopathy, and 12.1% for CHD.

**Table 1:** Demographic and clinical characteristics of the 273 subjects with type 2 diabetes of the TESEO study.

**Variables**	**Study population *n* = 273**
Age (years)	61.04 ± 8.1
Male gender (%)	48.7
**Smoking status (%)**	
No smokers	40.7
Ex-smokers	40.7
Active smokers	18.7
**Physical activity (%)**	
Low	46.2
Intermediate	44.7
High	9.2
MDS (*n*)	8.04 ± 1.94
BMI (Kg/m^2^)	33.10 ± 5.80
Obesity (BMI ≥ 30; %)	70
Diabetes duration (years)	3.90 ± 5.35
HbA1c (%)	6.58 ± 1.00
Hypertension (%)	93.4
Systolic BP (mmHg)	135.6 ± 16.9
Diastolic BP (mmHg)	84.1 ± 9.8
Total cholesterol (mg/dl)	173 ± 43
LDL cholesterol (mg/dl)	96 ± 37
HDL cholesterol (mg/dl)	48 (40–56)
Triglycerides (mg/dl)	123 (92–163)

Data are expressed as mean ± SD, percentage or median (25 °-75 ° percentile) for non-normally distributed data. BMI, body mass index; BP, blood pressure; HbA1c, glycated hemoglobin; HDL, high-density lipoprotein; LDL, low-density lipoprotein; MDS, Mediterranean diet score.

### 3.2 Soft drink intake

Overall, SDs consumption was low. More than half of the participants (53.5%) reported rarely or never consuming SDs, 35.5% consumed 1–4 servings per month, 9.9% consumed >1 servings per week, and only 1.1% reported >1 serving/day. Two highest SDs intake categories were thus combined. Table 2 presents the demographic, anthropometric, and clinical characteristics stratified by SDs consumption. Age, adherence to the MedDiet, total calorie intake, diabetes duration, BMI, and HDL cholesterol levels differed significantly across groups. No significant differences were observed in level of physical activity, smoking status, HbA1c, triglycerides, hypertension, or total and LDL cholesterol across SDs intake categories.

**Table 2:** Demographic, anthropometric, and clinical characteristics of the study population stratified by soft drinks consumption.

**Variables**	**Soft drinks consumption**			***p*-value**
	**Rarely/never**	**1–4 servings/month**	> **1 serving/week**	
*N*.	146	97	30	
Age (years)	62.0 ± 8.8	61.0 ± 6.8	56.5 ± 7.2	**0.003**
Male gender (%)	47.9	44.3	66.7	0.098
**Smoking status (%)**				
No smokers	43.2	41.2	26.7	0.198
Ex-smokers	41.8	35.1	53.3	
Active smokers	15.1	23.7	20.0	
**Physical activity (%)**				
Low	41.8	53.6	43.3	0.409
Intermediate	48.6	37.1	50.0	
High	9.6	9.3	6.7	
Total caloric intake (kcal/day)	1,880 (1,629–2,410)	2,167 (1,833–2,597)	2,671 (2,038–3,367)	**< 0.001**
MDS (*n*)	8.56 ± 1.94	7.62 ± 1.72	6.87 ± 1.78	**< 0.001**
BMI (Kg/m^2^)	32.3 ± 5.22	33.18 ± 6.18	36.85 ± 5.92	**< 0.001**
Obesity (BMI ≥ 30; %)	68.5	67.0	86.7	0.107
Diabetes duration (years)	4.66 ± 6.11	3.16 ± 4.27	2.57 ± 3.82	**0.035**
HbA1c (%)	6.64 ± 1.11	6.49 ± 0.84	6.59 ± 0.91	0.515
Hypertension (%)	93.8	91.8	96.7	0.570
Total cholesterol (mg/dl)	172 ± 46	175 ± 40	172 ± 44	0.838
LDL cholesterol (mg/dl)	95 ± 39	98 ± 35	94 ± 39	0.810
HDL cholesterol (mg/dl)	49 (41–58)	48 (40–57)	41 (38–50)	**0.031**
Triglycerides (mg/dl)	125 (88–162)	114 (97–154)	131 (88–203)	0.394

Data are expressed as mean ± SD, percentage or median (25 °-75 ° percentile) for non-normally distributed data. BMI, body mass index; HbA1c, glycated hemoglobin; HDL, high-density lipoprotein; LDL, low-density lipoprotein; MDS, Mediterranean diet score. Statistically significant *p*-values are reported in bold.

### 3.3 Liver fibrosis

The median CAP and LSM values in the study population were 308.0 (279.0–341.0) dB/m and 5.2 (4.4–6.0) kPa, respectively. Significant liver fibrosis was present in 13.6% of participants. Table 3 summarizes demographic, anthropometric, and clinical characteristics according to fibrosis status. Participants with fibrosis had a less favorable metabolic profile, with a higher prevalence of obesity, higher total calorie intake and lower HDL cholesterol levels. By contrast, no significant differences were observed in HbA1c, total and HDL cholesterol, triglycerides, and prevalence of hypertension. As expected, AST, ALT, and CAP values were significantly higher among participants with liver fibrosis.

**Table 3:** Demographic, anthropometric, and clinical characteristics of the study population stratified by fibrosis status.

**Variables**	**LSM ≤ 7 kPa**	**LSM >7 kPa**	***p*-value**
*N*°	236	37	
Sex M (%)	47.5	56.8	0.191
Age (years)	61.4 ± 8.0	59.1 ± 8.3	0.119
**Physical activity (%)**			
Low	44.5	56.8	0.393
Intermediate	46.2	35.1	
High	9.3	8.1	
**Smoking status (%)**			
Never smokers	40.3	43.2	0.778
Ex-smokers	41.5	35.1	
Active smokers	18.2	21.6	
Total calorie intake (kcal/day)	2,024 (1,707–2,564)	2,213 (1,886–2,935)	**0.032**
MDS (*n*)	8.11 ± 1.98	7.59 ± 1.55	0.066
**Soft drink consumption**			
Never/rarely	56.4	35.1	**0.007**
1–4 servings/month	34.7	40.5	
>1 serving/week	8.9	24.3	
Obesity (BMI ≥ 30; %)	66.9	89.2	**0.006**
Hypertension (%)	93.6	91.9	0.720
Diabetes duration (years)	3.73 ± 5.07	4.98 ± 6.86	0.184
HbA1c (%)	6.58 ± 1.04	6.62 ± 0.73	0.819
Total cholesterol (mg/dl)	174 ± 44	169 ± 41	0.520
LDL cholesterol (mg/dl)	96 ± 38	95 ± 35	0.843
HDL cholesterol (mg/dl)	49 (40–58)	42 (36–54)	**0.025**
Triglycerides (mg/dl)	124 (89–168)	122 (105–152)	0.390
AST (IU/L)	20.5 (17–25)	26 (21–32.5)	**< 0.001**
ALT (IU/L)	23 (17–31)	30 (20–44)	**0.003**
CAP (dB/m)	305 (276–337)	336 (319–356)	**< 0.001**

Data are expressed as mean ± SD, percentage or median (25 °-75 ° percentile) for non-normally distributed data. ALT, alanine aminotransferase; AST, aspartate aminotransferase; BMI, body mass index; CAP, controlled attenuation parameter; HbA1c, glycated hemoglobin; HDL, high-density lipoprotein; LDL, low-density lipoprotein; LSM, liver stiffness measurement; MDS, Mediterranean diet score. Statistically significant *p*-values are reported in bold.

SDs consumption also differed significantly by fibrosis status, primarily driven by the subgroup consuming ≥2 servings per week, which was more prevalent among participants with fibrosis (24.3%) compared with those without (8.9%).

### 3.4 Linear regression analysis

In the age- and sex-adjusted linear regression analysis, soft drink intake was positively associated with log-LSM (β = 0.181; 95% CI: 0.062–0.299; *p* = 0.003).

In the multivariate model, this association remained significant after additional adjustment for diabetes duration, log-total caloric intake, BMI, and log-HDL (β = 0.153; 95% CI: 0.030–0.270; *p* = 0.014). Besides SDs intake, variables significantly associated with log-LSM were diabetes duration (β = 0.133; 95% CI:0.020–0.246; *p* = 0.022) and BMI (β = 0.301; CI: 0.189–0.412; *p* < 0.001). BMI and CAP were modeled separately to prevent collinearity, and results remained consistent when BMI was replaced by log-CAP (β = 0.150; 95% CI: 0.028–0.274; *p* = 0.017).

### 3.5 Logistic regression analysis

Multivariable logistic regression analyses were performed to evaluate whether classes of SDs consumption were independently associated with liver stiffness after adjustment for potential risk factors and confounders. High soft drink intake (>1 serving/week) was associated with increased odds [OR: 3.77; 95% CI: 1.33–10.66] of liver stiffness (LSM >7) compared to subjects never/rarely assuming SDs, after adjustment for age, sex, diabetes duration, and obesity (Table 4). A sensitivity analysis applying the LSM ≥8 kPa threshold yielded consistent results (OR: 5.07; 95% CI 1.52–16.90; Supplementary Table S1). Inclusion into the model of tertile of CAP in place of obesity did not modify the results (LSM >7 OR: 3.11 95% CI: 1.09–8.86).

**Table 4:** Logistic regression: association between frequency of soft drinks consumption and LSM>7 kPa.

**Variables**	**LSM>7 kPa OR (CI 95%)**	**LSM>7 kPa OR (CI 95%)**
**Soft drink consumption**		
Never/rarely	1	1
1–4 servings/month	2.18 (0.94–5.02)	2.19 (0.95–5.08)
>1 serving/week	3.77 (1.33–10.66)	3.11 (1.09–8.86)
Age (years)	0.97 (0.93–1.02)	0.98 (0.93–1.03)
Sex (M)	1.55 (0.74–3.26)	1.49 (0.70–3.15)
Diabetes duration (years)	1.07 (1.01–1.11)	1.07 (1.00–1.15)
Obesity (y/n)	4.11 (1.36–12.42)	–
**CAP**		
Lower tertile	–	1
Intermediate tertile		5.06 (1.36–18.82)
Higher tertile		7.49 (2.06–27.18)

CAP, controlled attenuation parameter; CI, confidence interval; LSM, liver stiffness measurement; OR, odds ratio. CAP tertiles: lower 248–292 db/m; intermediate 293–329 db/m; higher 330–400 db/m.

## 4 Discussion

In this cross-sectional study of adults with T2DM, we found a strong and independent association between SDs consumption and liver stiffness, as measured by transient elastography, suggesting that SDs intake may contribute to an increased risk of liver fibrosis in this high-risk population.

In multivariable linear regression analyses, the frequency of SDs intake was positively associated with liver stiffness after adjustment for multiple confounders, including total caloric intake, BMI, and CAP. Consistently, in logistic regression, participants consuming >1 serving/week of SDs had a 3.77-fold higher odds of significant liver fibrosis compared with those who rarely or never consumed SDs, independent of obesity and diabetes duration. Notably, the association was even stronger when applying a stricter fibrosis threshold (LSM ≥8.0 kPa) that identifies individuals at moderate-to-high risk of advanced fibrosis (27). Since only 9.2% of participants had LSM ≥8.0 kPa, this result should be interpreted cautiously; nonetheless, the consistent directionality across thresholds supports the robustness of the association.

SD consumption has been consistently linked to MASLD in both cross-sectional and prospective studies (1, 14, 17, 28). This relationship is independent of metabolic syndrome (16) and is observed even for “diet SDs” (29). Recently, higher consumption of sugar-sweetened beverages was also associated with increased liver stiffness in healthy adults (18). To our knowledge, however, this is the first study to explore this association between SD consumption and liver fibrosis specifically in individuals with T2DM - a group at particularly high risk for progression of MASLD.

Our findings suggest that SDs may promote hepatic fibrogenesis through mechanisms extending beyond fat accumulation and metabolic dysregulation. The biological plausibility of this association is supported by experimental evidence showing that fructose-rich beverages enhance *de novo* lipogenesis, hepatic fat deposition, and oxidative stress—pathways that can accelerate hepatocellular injury and fibrogenesis (30, 31).

Overall, SDs consumption was low, with only 1.1% of participants reporting intake of more than one serving per week. However, SDs consumption in the general Italian population of a similar age range is also relatively low compared with that of other European countries (32) and of the United States (US) (33). The relatively low SDs exposure in this Mediterranean cohort provides a unique setting, illustrating that adverse associations can arise even at lower consumption levels than those typically reported in North American populations. SDs consumption is a negative component of the MedDiet score (20), therefore the MedDiet score was not included as a covariate in our regression models to avoid multicollinearity. Nevertheless, recent evidence indicates that greater adherence to the MedDiet is independently associated with a lower risk of liver fibrosis (34–36), and our findings suggest that this protective effect may be partly mediated by reduced SDs consumption.

Significant fibrosis was relatively uncommon, likely reflecting the short duration of T2DM in this cohort. Interestingly, participants with significant fibrosis showed no differences in HbA1c, triglycerides, total cholesterol, or blood pressure, suggesting that traditional metabolic markers may not fully capture the risk of hepatic injury. Notably, in the logistic model, the association between intermediate SDs intake and liver fibrosis did not reach statistical significance compared with non-consumers, possibly reflecting either a threshold effect or limited power to detect smaller effects at lower exposure levels.

This study has several strengths. Participants were well-characterized in terms of anthropometric, metabolic, and clinical parameters. SDs consumption was assessed using the validated EPIC food frequency questionnaire, which captures total energy intake and allows for adjustment by caloric intake, thereby reducing residual confounding. Liver fibrosis was evaluated using VCTE, a well-validated, non-invasive technique that shows strong correlation with histological fibrosis and predicts both liver-related and overall mortality (37, 38). This method was preferred to surrogate indices such as the NFS or FIB-4, which have lower accuracy (39). Moreover, since BMI is a component of the NFS and age is included in both NFS and FIB-4 (5), adjusting for these variables as potential confounders is inherently limited when using such composite scores. Although sample size was relatively small, given the limited number of studies investigating hepatic fibrosis, our results address an important knowledge gap in MASLD epidemiology.

However, several limitations should be acknowledged. First, the cross-sectional design precludes causal inference. Second, SDs intake was self-reported and assessed using a single question. However, dietary data were collected by trained dietitians rather than through self-administration, further enhancing accuracy and reliability. Moreover, any misclassification would probably attenuate rather than exaggerate observed associations. Third, the relatively small number of daily SDs consumers and of people with significant liver fibrosis limited statistical power. Fourth, liver steatosis was diagnosed using CAP, which performs well in detecting fatty liver, but optimal thresholds vary widely (40) meaning some cases of mild steatosis with elevated LSM may have been missed. Finally, liver fibrosis was not histologically confirmed, and thus some degree of misclassification cannot be excluded—although this would tend to bias results toward the null.

In conclusion, this study provides novel evidence that SDs consumption is independently associated with increased liver stiffness in individuals with T2DM and MASLD. The results reinforce current dietary recommendations to limit SDs intake as part of comprehensive lifestyle strategies aimed at preventing or slowing MASLD progression in patients with diabetes. Prospective and interventional studies are warranted to confirm causality and clarify whether reducing SDs consumption can attenuate fibrosis progression over time.

## Data Availability

The raw data supporting the conclusions of this article will be made available by the authors, without undue reservation.

## References

[1] Younossi Z Anstee QM Marietti M Hardy T Henry L Eslam M . Global burden of NAFLD and NASH: trends, predictions, risk factors and prevention. Nat Rev Gastroenterol Hepatol. (2018) 15:11–20. doi: 10.1038/nrgastro.2017.109 28930295

[2] Karlsen TH Sheron N Zelber-Sagi S Carrieri P Dusheiko G Bugianesi E . The EASL–lancet liver commission: protecting the next generation of Europeans against liver disease complications and premature mortality. Lancet. (2022) 399:61–116. doi: 10.1016/S0140-6736(21)01701-3 34863359

[3] Younossi ZM Kalligeros M Henry L. Epidemiology of metabolic dysfunction-associated steatotic liver disease. Clin Mol Hepatol. (2025) 31:32–50. doi: 10.3350/cmh.2024.0431 39159948 PMC11925440

[4] Le P Tatar M Dasarathy S Alkhouri N Herman WH Taksler GB . Estimated burden of metabolic dysfunction-associated steatotic liver disease in US adults, 2020 to 2050. JAMA Netw Open. (2025) 8:54707–21. doi: 10.1001/jamanetworkopen.2024.54707 39821400 PMC11742522

[5] Tacke F Horn P Wong VWS Ratziu V Bugianesi E Francque S . EASL-EASD-EASO clinical practice guidelines on the management of metabolic dysfunction-associated steatotic liver disease (MASLD). Obes Facts. (2024) 17:374–444. doi: 10.1159/000539371 38852583 PMC11299976

[6] Ekstedt M Hagström H Nasr P Fredrikson M Stål P Kechagias S . Fibrosis stage is the strongest predictor for disease-specific mortality in NAFLD after up to 33 years of follow-up. Hepatology. (2015) 61:1547–54. doi: 10.1002/hep.27368 25125077

[7] Younossi ZM Golabi P Price JK Owrangi S Gundu-Rao N Satchi R . The global epidemiology of nonalcoholic fatty liver disease and nonalcoholic steatohepatitis among patients with type 2 diabetes. Clin Gastroenterol Hepatol. (2024) 22:1999–2010.e8. doi: 10.1016/j.cgh.2024.03.006 38521116

[8] Caviglia GP Ferro A D'Ambrosio R Perbellini R Lampertico P Periti G . Effectiveness of a model of care based on fibrosis-4 and liver stiffness measurement for the screening of patients with type 2 diabetes mellitus at risk of advanced liver disease: results from an Italian prospective multicentre study. Am J Gastroenterol. (2025). doi: 10.14309/ajg.0000000000003493 40226934

[9] Huang DQ Wilson LA Behling C Kleiner DE Kowdley KV Dasarathy S . Fibrosis progression rate in biopsy-proven nonalcoholic fatty liver disease among people with diabetes versus people without diabetes: a multicenter study. Gastroenterology. (2023) 165:463–72.e5. doi: 10.1097/HEP.0000000000001015 37127100 PMC10699569

[10] Jensen T Abdelmalek MF Sullivan S Nadeau KJ Green M Roncal C . Fructose and sugar: a major mediator of non-alcoholic fatty liver disease. J Hepatol. (2018) 68:1063–75. doi: 10.1016/j.jhep.2018.01.019 29408694 PMC5893377

[11] Qin P Li Q Zhao Y Chen Q Sun X Liu Y . Sugar and artificially sweetened beverages and risk of obesity, type 2 diabetes mellitus, hypertension, and all-cause mortality: a dose-response meta-analysis of prospective cohort studies. Eur J Epidemiol. (2020) 35:655–71. doi: 10.1007/s10654-020-00655-y 32529512

[12] Narain A Kwok CS Mamas MA. Soft drinks and sweetened beverages and the risk of cardiovascular disease and mortality: a systematic review and meta-analysis. Int J Clin Pract. (2016) 70:791–805. doi: 10.1111/ijcp.12841 27456347

[13] Mullee A Romaguera D Pearson-Stuttard J Viallon V Stepien M Freisling H . Association between soft drink consumption and mortality in 10 European countries. JAMA Intern Med. (2019) 179:1479–90. doi: 10.1001/jamainternmed.2019.2478 31479109 PMC6724165

[14] Naomi ND Ngo J Brouwer-Brolsma EM Buso MEC Soedamah-Muthu SS Pérez-Rodrigo C . Sugar-sweetened beverages, low/no-calorie beverages, fruit juice and non-alcoholic fatty liver disease defined by fatty liver index: the SWEET project. Nutr Diabetes. (2023) 13:6. doi: 10.1038/s41387-023-00237-3 37085478 PMC10121594

[15] Zhang S Gu Y Bian S Lu Z Zhang Q Liu L . Soft drink consumption and risk of nonalcoholic fatty liver disease: results from the Tianjin chronic low-grade systemic inflammation and health (TCLSIH) cohort study. Am J Clin Nutr. (2021) 113:1265–74. doi: 10.1093/ajcn/nqaa380 33564868

[16] Abid A Taha O Nseir W Farah R Grosovski M Assy N. Soft drink consumption is associated with fatty liver disease independent of metabolic syndrome. J Hepatol. (2009) 51:918–24. doi: 10.1016/j.jhep.2009.05.033 19765850

[17] Schwimmer JB Ugalde-Nicalo P Welsh JA Angeles JE Cordero M Harlow KE . Effect of a low free sugar diet vs usual diet on nonalcoholic fatty liver disease in adolescent boys: a randomized clinical trial. JAMA. (2019) 321:256–65. doi: 10.1001/jama.2018.20579 30667502 PMC6440226

[18] Leung CW Tapper EB. Sugar-sweetened beverages are associated with increased liver stiffness and steatosis among apparently healthy adults in the United States. Clin Gastroenterol Hepatol. (2022) 20:959–61.e1. doi: 10.1016/j.cgh.2021.05.052 34058408 PMC8627516

[19] Day N Oakes S Luben R Khaw KT Bingham S Welch A . EPIC-Norfolk: study design and characteristics of the cohort. European prospective investigation of cancer. Br J Cancer. (1999) 80 Suppl 1:95–103. 10466767

[20] García-Conesa MT Philippou E Pafilas C Massaro M Quarta S Andrade V . Exploring the validity of the 14-item Mediterranean diet adherence screener (Medas): a cross-national study in seven European countries around the Mediterranean region. Nutrients. (2020) 12:1–18. doi: 10.3390/nu12102960 32992649 PMC7601687

[21] Rinella ME Lazarus JV Ratziu V Francque SM Sanyal AJ Kanwal F . A multisociety Delphi consensus statement on new fatty liver disease nomenclature. J Hepatol. (2023) 79:1542–56. doi: 10.1097/HEP.0000000000000696 37364790

[22] Craig CL Marshall AL Sjostrom M Bauman AE Booth ML Ainsworth BE . International physical activity questionnaire: 12-country reliability and validity. Med Sci Sports Exerc. (2003) 35:1381–95. doi: 10.1249/01.MSS.0000078924.61453.FB 12900694

[23] ElSayed NA McCoy RG Aleppo G Balapattabi K Beverly EA Briggs Early K . 10. cardiovascular disease and risk management: standards of care in diabetes-−2025. Diabetes Care. (2025) 48:S207–38. doi: 10.2337/dc25-S010 39651970 PMC11635050

[24] ElSayed NA McCoy RG Aleppo G Balapattabi K Beverly EA Briggs Early K . 11. chronic kidney disease and risk management: standards of care in diabetes −2025. Diabetes Care. (2025) 48:S239–51. doi: 10.2337/dc25-S011 39651975 PMC11635029

[25] FETA EPIC software. Available online at: https://www.mrc-epid.cam.ac.uk/research/measurement-platform/dietary-assessment/feta/ (Accessed December 10, 2025).

[26] EASL-ALEH clinical practice guidelines: non-invasive tests for evaluation of liver disease severity and prognosis. J Hepatol. (2015) 63:237–64. doi: 10.1016/j.jhep.2015.04.006 25911335

[27] Wong VW-S Vergniol J Wong GL-H Foucher J Chan HL-Y Le Bail B . Diagnosis of fibrosis and cirrhosis using liver stiffness measurement in nonalcoholic fatty liver disease. Hepatology. (2010) 51:454–62. doi: 10.1002/hep.23312 20101745

[28] Assy N Nasser G Kamayse I Nseir W Beniashvili Z Djibre A . Soft drink consumption linked with fatty liver in the absence of traditional risk factors. Can J Gastroenterol. (2008) 22:811–6. doi: 10.1155/2008/810961 18925303 PMC2661299

[29] Wu Y Tan Z Zhen J Liu C Zhang J Liao F . Association between diet soft drink consumption and metabolic dysfunction-associated steatotic liver disease: findings from the NHANES. BMC Public Health. (2023) 23:2286–93. doi: 10.1186/s12889-023-17223-0 37985986 PMC10658943

[30] Stanhope KL Schwarz JM Keim NL Griffen SC Bremer AA Graham JL . Consuming fructose-sweetened, not glucose-sweetened, beverages increases visceral adiposity and lipids and decreases insulin sensitivity in overweight/obese humans. J Clin Invest. (2009) 119:1322–34. doi: 10.1172/JCI37385 19381015 PMC2673878

[31] Abdelmalek MF Suzuki A Guy C Unalp-Arida A Colvin R Johnson RJ . Increased fructose consumption is associated with fibrosis severity in patients with nonalcoholic fatty liver disease. Hepatology. (2010) 51:1961–71. doi: 10.1002/hep.23535 20301112 PMC2922495

[32] Eurostat Data Browser. Frequency of Drinking Sugar-Sweetened Soft Drinks by Sex, Age, and Educational Attainment Level. (2022). Available online at: https://ec.europa.eu/eurostat/databrowser/view/hlth_ehis_fv7e/default/table?lang=en (Accessed December 10, 2025).

[33] Dai J Soto MJ Dunn CG Bleich SN. Trends and patterns in sugar-sweetened beverage consumption among children and adults by race and/or ethnicity, 2003–2018. Public Health Nutr. (2021) 24:2405–10. doi: 10.1017/S1368980021001580 33843567 PMC10195631

[34] Castelnuovo G Perez-Diaz-Del-Campo N Rosso C Guariglia M Armandi A Nicolosi A . Impact of chronotype and Mediterranean diet on the risk of liver fibrosis in patients with non-alcoholic fatty liver disease. Nutrients. (2023) 15:3257–69. doi: 10.3390/nu15143257 37513675 PMC10385040

[35] Perez-Diaz-Del-Campo N Castelnuovo G Rosso C Nicolosi A Guariglia M Dileo E . Impact of health related QoL and Mediterranean diet on liver fibrosis in patients with NAFLD. Nutrients. (2023) 15:3018–27. doi: 10.3390/nu15133018 37447344 PMC10346905

[36] Sualeheen A Tan S-Y Daly RM Georgousopoulou E Roberts SK George E-S. Higher diet quality is associated with a lower prevalence of MASLD and adverse health outcomes: insights from NHANES 2005 to 2020. Eur J Nutr. (2025) 64:289. doi: 10.1007/s00394-025-03809-4 41051618 PMC12500818

[37] Lin H Lee HW Yip TC-F Tsochatzis E Petta S Bugianesi E . Vibration-controlled transient elastography scores to predict liver-related events in steatotic liver disease. JAMA. (2024) 331:1287. doi: 10.1001/jama.2024.1447 38512249 PMC10958386

[38] Boursier J Vergniol J Guillet A Hiriart J-B Lannes A Le Bail B . Diagnostic accuracy and prognostic significance of blood fibrosis tests and liver stiffness measurement by FibroScan in non-alcoholic fatty liver disease. J Hepatol. (2016) 65:570–8. doi: 10.1016/j.jhep.2016.04.023 27151181

[39] Pennisi G Enea M Falco V Aithal GP Palaniyappan N Yilmaz Y . Noninvasive assessment of liver disease severity in patients with nonalcoholic fatty liver disease (NAFLD) and type 2 diabetes. Hepatology. (2023) 78:195–211. doi: 10.1097/HEP.0000000000000351 36924031

[40] Cao Y Xiang L Qi F Zhang Y Chen Y Zhou X. Accuracy of controlled attenuation parameter (CAP) and liver stiffness measurement (LSM) for assessing steatosis and fibrosis in non-alcoholic fatty liver disease: a systematic review and meta-analysis. EClinicalMedicine. (2022) 51:101547. doi: 10.1016/j.eclinm.2022.101547 35844772 PMC9284399

