# Supplementary material for: Soft drink consumption and liver fibrosis in type 2 diabetes
Source: Front Nutr. 2025 Dec 18;12:1726040. doi: 10.3389/fnut.2025.1726040 (PMC12756108; doi:10.3389/fnut.2025.1726040)
Supplement: Supplementary file 1 [file Table_1.docx]

Supplementary Material

**Supplementary Table S1. Logistic regression: association between frequency of soft drinks consumption and LSM>8 kPa**

|  | **LSM>8 kPa**  **OR (CI 95%)** | **LSM>8 kPa**  **OR (CI 95%)** |
| --- | --- | --- |
| **Soft drink consumption**  Never/rarely  1-4 servings/month  >1 serving/week | 1  2.39 (0.86-6.63)  5.07 (1.52-16.90) | 1  2.38 (0.86-6.58)  4.23 (1.23-14.24) |
| Age (years) | 0.98 (0.93-1.04) | 0.98 (0.93-1.04) |
| Sex (M) | 1.40 (0.58-3.37) | 1.34 (0.55-3.23) |
| Diabetes duration (years) | 1.08 (1.01-1.16) | 1.08 (1.00-1.17) |
| Obesity (y/n) | 3.32 (0.92-11.91) | - |
| CAP  Lower tertile  Intermediate tertile  Higher tertile | - | 1  5.10 (1.05-24.84)  6.26 (1.31-29.85) |

CAP, Controlled Attenuation Parameter; CI, confidence interval; LSM, Liver Stiffness Measurement; OR, odds ratio. CAP tertiles: lower 248-292 db/m; intermediate 293-329 db/m; higher 330-400 db/m.
